# Using Twitter to Understand the COVID-19 Experiences of People With Dementia: Infodemiology Study

**DOI:** 10.2196/26254

**Published:** 2021-02-03

**Authors:** Juanita-Dawne Bacsu, Megan E O'Connell, Allison Cammer, Mahsa Azizi, Karl Grewal, Lisa Poole, Shoshana Green, Saskia Sivananthan, Raymond J Spiteri

**Affiliations:** 1 Department of Psychology University of Saskatchewan Saskatoon, SK Canada; 2 College of Pharmacy and Nutrition University of Saskatchewan Saskatoon, SK Canada; 3 Department of Computer Science University of Saskatchewan Saskatoon, SK Canada; 4 Dementia Advocacy Canada Calgary, AB Canada; 5 Alzheimer Society of Canada Toronto, ON Canada

**Keywords:** Twitter, social media, dementia, COVID-19, health policy, experience, support, disorder, theme, collaborate, quality of life

## Abstract

**Background:**

The COVID-19 pandemic is affecting people with dementia in numerous ways. Nevertheless, there is a paucity of research on the COVID-19 impact on people with dementia and their care partners.

**Objective:**

Using Twitter, the purpose of this study is to understand the experiences of COVID-19 for people with dementia and their care partners.

**Methods:**

We collected tweets on COVID-19 and dementia using the GetOldTweets application in Python from February 15 to September 7, 2020. Thematic analysis was used to analyze the tweets.

**Results:**

From the 5063 tweets analyzed with line-by-line coding, we identified 4 main themes including (1) separation and loss; (2) COVID-19 confusion, despair, and abandonment; (3) stress and exhaustion exacerbation; and (4) unpaid sacrifices by formal care providers.

**Conclusions:**

There is an imminent need for governments to rethink using a one-size-fits-all response to COVID-19 policy and use a collaborative approach to support people with dementia. Collaboration and more evidence-informed research are essential to reducing COVID-19 mortality and improving the quality of life for people with dementia and their care partners.

## Introduction

The COVID-19 pandemic is having an impact on people with dementia. In Canada, almost two-thirds of all COVID-19–related deaths have been people with dementia [1]. In the United Kingdom, 50% of COVID-19–related deaths in care homes have been people with dementia [2].

In comparison to other groups, people with dementia are among the most vulnerable to the COVID-19 pandemic [3]. Although people with dementia are not necessarily more susceptible to COVID-19, advancing age, frailty, and coexisting health conditions often associated with dementia (eg, cardiovascular disease, hypertension, or diabetes) increase the risk of complications [4,5]. Moreover, social isolation measures (eg, stay-at-home orders, visitation bans, and lockdowns in care facilities) from COVID-19 may increase the risk of hospitalization and mortality for people with dementia [6].

People with dementia also face a heightened risk of COVID-19 exposure due to cognitive impairment and memory loss [3]. For example, people with dementia may have challenges remembering or understanding self-protection protocols such as wearing a mask, using proper hand hygiene, and maintaining physical distance from others [7,8]. Despite these challenges, there is a paucity of knowledge on the COVID-19 pandemic’s impact on people with dementia.

There is an urgent need to understand the experiences of people with dementia and their care partners in the COVID-19 pandemic [8-10]. Mitigation strategies that include physical distancing make it difficult to conduct timely and collaborative research, with many universities suspending recruitment and in-person studies during the pandemic [11]. Given these difficulties, examining the impact of COVID-19 requires ingenuity and innovation.

With over 330 million monthly users [12], the microblogging and social networking website Twitter [13] presents an innovative opportunity to examine the COVID-19 impact on people with dementia. For example, Twitter users are publicly sharing lived experiences of COVID-19 and dementia. The purpose of this study is to use Twitter to understand the COVID-19 experiences of people with dementia and their care partners.

## Methods

### Recruitment

We scraped tweets posted in the English language containing synonyms for dementia (eg, “Alzheimer’s disease” and “Lewy Body disease”) and synonyms for COVID-19 (including nontechnical phrases such as “Corona”) during the period from February 15 to September 7, 2020, from Twitter using the GetOldTweets application programming interface in Python [14]. The Tweets were not geo-tagged.

Twitter is a social media and microblogging website where users share their posts with the public. Following on existing studies, there is a general consensus that tweets posted publicly on Twitter can be used for research [15,16]. Tweets on the Twitter website are located within the public domain; informed consent was not obtained.

### Data Exclusion

From the approximately 20,800 tweets that were gathered using these criteria, we applied filters referring to candidates for the US presidential election (eg, synonyms for “Donald Trump” and “Joe Biden”). In addition, the filter “Tom Seaver“ was used to delete tweets of public reactions to the major league baseball player who was reported to have died on August 31, 2020, of complications due to COVID-19 and dementia with Lewy bodies. Retweets and nonoriginal tweets were also excluded. Finally, to increase the likelihood of scraping tweets that described personal experiences with dementia during the era of COVID-19, we excluded tweets that did not include synonyms for familial relationships (eg, “father,” “mother,” and “grandparent”) or friends and acquaintances (eg, “buddy,” “pal,” and “neighbour”). This filtering procedure resulted in a total of 5063 tweets that were extracted into an Excel (Microsoft Corporation) spreadsheet for qualitative line-by-line coding as the basis for thematic analysis.

### Data Analysis: Coding and Intercoder Consistency

To develop a robust codebook, authors JDB and MEO read and reread 300 tweets. The researchers independently assigned codes to each of the 300 tweets. After coding the tweets, the two researchers met to discuss their code lists and developed an initial codebook. The initial codebook consisted of 18 codes with code definitions, keywords, and specific examples (eg, tweets).

To test intercoder consistency, a team of six researchers pilot-tested 100 tweets by independently coding the tweets according to the codebook. Once the team finished their coding, the codes were compared with a coding example sheet that JDB and MEO developed. Following this pilot test, a group coding workshop was held to collectively pilot-code 50 tweets and discuss any inconsistencies in the interpretation of the codes. During this workshop, group discussion resolved coding uncertainties and disputes, leading to the refinement of the codebook (eg, overlapping codes or unused codes). The final version of the codebook consisted of 9 codes including (1) death, (2) fear for person with dementia’s health and well-being, (3) challenges and unmet needs, (4) separation or restricted visiting, (5) formal care provider challenges, (6) supports described, (7) informal caregiver’s health and well-being, (8) stories of survival, and (9) user identifies as a person with dementia (used as a second tag vs a primary code).

The tweets (n=5063) were then divided among the seven authors (approximately 723 tweets each) for descriptive analysis, with JDB managing intercoder consistency throughout the coding process. For 7 consecutive days, each coder completed approximately 100 codes per day and sent them to JDB. Coders flagged any tweets of which they were unsure or uncertain for JDB to review. In addition to reviewing the flagged tweets, the JDB randomly reviewed 10% of each coder’s work and provided feedback (eg, flagged tweets or any inconsistencies) for each day of coding. Throughout the coding process, JDB worked in direct collaboration with the MEO to discuss any discrepancies or uncertainties within the data. Once the coding was completed, two team meetings were held to identify and discuss the key themes arising from the data.

## Results

From the 5063 tweets analyzed, we identified 4 main themes including (1) separation and loss; (2) COVID-19 confusion, despair, and abandonment; (3) stress and exhaustion; and (4) risks of exposure and personal sacrifices faced by formal care providers. Illustrative tweets are provided and are unedited for grammar.

### Separation and Loss

This theme captures the psychological sense of loss due to physical barriers that create separation. Many personal descriptions included discussion of the physical separation, which includes separation due to death, separation during the process of dying during the COVID-19 pandemic, and separation due to visitation bans because of COVID-19. Underlying these descriptors of physical separation is a clear psychological disconnection accompanied by feelings of loss. Separation creates a psychological sense of loss. A notable subtheme in separation and loss is the expression of the sense of loss expressed by care partners at the acceleration of decline perceived in persons with dementia during COVID-19, which was frequently blamed on the visitation bans and imposed lockdowns in care homes and health facilities. The intersection of the multiple dimensions of loss and separation are highlighted in the following illustrative quotes:

Yesterday I lost my mom. Due to covid [sic] I was unable to see her for the last few months. I did get to FaceTime twice and we did chat on the phone until her dementia made it difficult for her to do so. There will be no memorial until it is cleared to do so. My heart is broken. 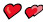

...My husband passed away a few days ago, victim of Covid [sic] protocol! He had dementia, didn’t understand why I couldn’t visit him. He lost hope, 36 lbs in 23 days; could not be saved. This is so cruel to do to our seniors/he was a veteran!!! WRONG!!!

...Dad had dementia and was otherwise healthy. He had daily visitors even though he had little memory of who they were. Covid [sic] closed the nursing home to visitors and he stopped eating. He lost 40lbs and died in June. It’s another aspect of the Covid [sic]. Dying Alone.

Let me tell you what this covid [sic] lockdown did, it killed my daddy. He had dementia and he was still doing good, then the lockdown, we weren't there to hold him and to help feed him. When we went to see him, he was a shell, there was nothing left of him. I am so angry.

### COVID-19 Confusion, Despair, and Abandonment

Another predominant theme was feelings of despair and abandonment among people with dementia from COVID-19 confinement and visitation bans. Many tweets described difficulties understanding COVID-19 displayed or experienced by persons with dementia and negative psychological consequences due to this confusion about COVID-19. Many reported that people with dementia could not understand the changes necessitated by the pandemic response; they required constant teaching, reminders, and reassurance. For some, this lack of understanding of COVID-19 led to challenges (described or implied) of living with the new policies for social distancing, mask wearing, and other sanitary precautions, which engendered feelings of hopelessness and despair. For many others, the lack of understanding the physical distancing restrictions due to COVID-19 led to feelings of abandonment and subsequent despair. For example, many described that their loved one could not understand why visits were no longer being made and why they could not have physical contact. The following tweets illustrate these issues:

Or live alone with dementia and all the trouble I have. I can't even drive myself to a doctor. I don't remember all the rules myself. I'm terrible at wearing a mask. Someone pointed out I had it inside out at the covid [sic] testing place. Im gonna [sic] die, I hope. Im [sic] tired of life.

You are told your Covid [sic] positive Mum is being discharged to you Covid [sic] negative dad. She has dementia. He is told to keep 2m away in their 3 bed bungalow. Is this NHS policy?

I’ve lost count of how many times I’ve been on the phone with my grandmother telling her that it’s not safe for her to leave the house because of COVID-19. Life with a loved one who has dementia right now is frustrating. Constantly re-teaching and remaining patient.

Hardest thing to hear is my mom trying to explain to my grandmother, who has Alzheimer and dementia, that we can’t see her because of the corona [sic]. My grandmother repeating that she is in jail. Asking where we are.

### Stress and Exhaustion Exacerbation

Care partners described stress and exhaustion (eg, mental, emotional, and physical) related to providing care for people with dementia in the context of the COVID-19 pandemic. Increased workload, disruption to routine, financial strain, mental health issues, social isolation, and loneliness were common features described.

COVID-19 confinement and lockdown measures substantially increased the workload of informal care partners by limiting or terminating access to support services such as day care programs, home care, respite, meal programs, medical specialists, and adequate care home options. As such, many described the difficulty of dealing with household chores, social isolation, and the increasing workload, which often led to feelings of mental, emotional, and physical exhaustion. Moreover, care partners described difficulties managing behavioral changes and worsening neuropsychiatric symptoms (eg, anxiety, agitation, anger, and depression) of their loved ones with dementia.

Care partners also discussed stress related to the fear of COVID-19 exposure and concern for the person with dementia’s health and well-being. This fear was especially apparent among care partners with loved ones staying in care homes or hospitals. Others noted that COVID-19 precautions were confusing to people with dementia and made appointments and outings more difficult not only for the person with dementia but also for the care partner. Some reported balancing decisions on whether to pursue health services based on how distressing the experience would be for their loved one (eg, due to being alone without care partner support). Many reported strain due to financial pressures related to losing work coupled with unease for the future of their loved one. Difficulty obtaining care services or relocation to long-term care due to the pandemic were noted. Further, some care partners described feeling a need to bring people with dementia home from long-term care to ensure their well-being. The following tweets highlight the challenges faced by informal care partners:

I've lost some income but the hardest part is being stuck 24/7 in the house with my Mom who has severe dementia. It is hell! She had a daycare but it closed. I can't get respite bc [sic] I'm terrified of exposing her to covid [sic]. I fight loneliness, depression and boredom everyday [sic]!

The services for people with dementia have had to fall into line with COVID rules and there are of course personal restrictions. A devastating combination for people who need safety, routine and gentle stimulation.

Another horrifying day. We are in isolation with my beloved 93 yo [sic] Mom. She has descended into terrifying hallucinations and extreme anger because of dementia. We can't get her into nursing care because of a Covid [sic] outbreak. Just getting thru [sic] each hour.

### Unpaid Sacrifices by Formal Care Providers

Formal care providers identified numerous sacrifices beyond their paid jobs. Formal care providers commonly expressed emotional connection to people with dementia and a sense of duty related to care, noting that this was more than simply a job. Formal care providers also described making personal sacrifices to work and provide care during the COVID-19 pandemic. For example, care providers discussed sacrificing their participation in family gatherings, parenting responsibilities, and social activities to help protect their patients and family members from potential exposure to the virus. Consequently, many expressed concern for the health of their families due to exposures at work and made trade-offs to help ensure the safety of their patients or residents, such as limiting their outside contacts. They also noted that caring for people with dementia involved a sacrifice of their own safety because of the lack of personal protective equipment (eg, gowns, gloves, masks, eye protection, and face shields), leading to stress of exposure. Finally, they reported their frustration with the reduced ability to support quality of life for people with dementia in the face of an increased workload due to COVID-19.

The workforce challenges experienced by formal care providers are captured in the following tweets:

People were discharged from hospital with covid [sic] and placed directly into my work. Trying to isolate a dementia patient with covid [sic] is impossible in a care home, they've already wandered down the corridor before we could even get our gloves on 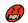

I'm a mental health nurse working in a dementia specialist nursing home. My fight is to keep corona [sic] out of the building. There are many of us who will be in hiding to protect our residents…

I’m a nurse with COVID, probably from reusing dirty N95s and working with dementia patients who simply could not grasp the need to wear a mask and social distance. I worked so hard to try not to get COVID.

## Discussion

### Principal Results

Using Twitter, the aim of this study is to understand the COVID-19 experiences of people with dementia and their care partners. People with dementia are among the most vulnerable to the pandemic in terms of exposure risk, social isolation, hospitalization, and mortality [4]. Understanding the impact of COVID-19 is urgently needed to reduce mortality and improve the quality of life for people with dementia and their care partners during the pandemic. Given the current COVID-19 context, Twitter provided a valuable means to support timely and innovative research during the pandemic.

In analyzing the 5063 tweets, this study found that people with dementia are experiencing substantial burden from the COVID-19 pandemic. For example, separation and institutional visiting bans were described as having a detrimental impact on people with dementia. Numerous tweets identified the effects of visitation bans on people with dementia, such as despair, loss, abandonment, social isolation, not eating, losing the will to live, and dying alone. Tweets also identified challenges faced by informal care partners, such as financial struggles, mental health issues, lack of formal supports, inadequate care home options, fear of COVID-19 exposure, and difficulties explaining COVID-19 (eg, quarantine, self-isolation, social distancing, and protective equipment) to people with dementia. In addition, tweets addressed workforce issues experienced by formal care providers, ranging from insufficient access to personal protective equipment to understaffing and having to sacrifice family responsibilities to provide formal care.

This study has significant implications for COVID-19 policy and practice. For example, findings from this study suggest that prohibitive visitation policies (eg, visitation bans and institutional lockdowns) in care homes and health facilities may not be advantageous or acceptable for people with dementia. Numerous tweets emphasized the issue of separation on mortality for people with dementia. Emerging data from the United Kingdom showed that, in care homes, more people have died from dementia than from COVID-19 [2]. Similarly, data from Canada [17,18] and the United States [1] mirror this trend, documenting thousands of excess dementia deaths in care homes throughout the pandemic. These findings suggest that secondary effects of the COVID-19 pandemic (eg, social isolation) may be causing rapid deterioration and mortality of people with dementia. Consequently, COVID-19 policies (eg, visitation bans and institutional lockdowns) that were initially intended to protect people from the virus may be causing significant harm to people with dementia. Accordingly, there is an imminent need for governments to rethink using a one-size-fits-all response to COVID-19 policy and use instead a collaborative approach to support people with dementia and their care partners. In moving forward, collaboration and partnerships with people with dementia are essential to developing targeted policies to protect people with dementia and their care partners.

Formal care providers desperately need additional resources (eg, allocated funding, personal protective equipment, adequate staffing, and mental health supports) to support people with dementia during the pandemic. Findings from this study identify a range of workforce challenges such as understaffing and lack of adequate personal protective equipment. Formal care providers in care homes and health facilities cannot provide optimal care and prevent COVID-19 exposure without having access to required resources and supports. Consequently, additional resources are needed to provide safeguards to protect both formal care providers and people with dementia during the pandemic.

Research is needed on the COVID-19 impact from the perspective of people with dementia and their care partners. Although several editorials, letters to the editor, and commentaries have discussed anticipated challenges of COVID-19 for people with dementia [8-10,19-21], few studies have involved people with dementia. Research is needed to examine the lived experiences of COVID-19 among people with dementia. Accordingly, more evidence-informed research is required to reduce mortality and understand the impact of COVID-19 on people with dementia and their care partners.

### Limitations

Twitter has a 280-character limit for each tweet. Given this limit, the user’s story and experience is confined to these restrictions. For example, important information and relevant details (eg, context, background, and confounding factors) may not be captured in the tweet. Consequently, qualitative interviews may provide a more comprehensive and in-depth perspective of the COVID-19 impact on people with dementia. In addition, given that Twitter is in the public domain, people may not feel comfortable sharing their full perspectives and lived experiences. Future research requires collaboration and partnerships with people with dementia to develop more in-depth knowledge on the impact of COVID-19 on people with dementia and their care partners.

In addition, there are some limitations related to the generalizability of our findings from Twitter. For example, no demographic information was collected in this study, and findings from Twitter may not be fully generalizable or representative of the general public living with dementia and their care partners (eg, age, gender, ethnicity, education, income, or employment background). However, existing data show that Twitter users are 56% male and 44% female, with the largest age groups of users being in the categories of 18-29 years (38%), 30-49 years (27%), and 50-64 years (17%) [22]. Since no demographic information was collected, another study limitation relates to sex and gender. Without any demographic information, it is impossible to make any inferences or draw specific conclusions regarding the impact of COVID-19 in relation to sex or gender. As such, more research is needed to examine the impact of COVID-19 in relation to the sex and gender of people with dementia and their care partners.

A final limitation of these data is the cross-sectional nature of our analysis and geographically blind nature of the data. It is possible that experiences of people with dementia and their care partners will vary over time, particularly over waves of the pandemic. We believe these current data captured what might be described as wave one of the pandemic. It is likely, however, that any temporal variability in experiences with COVID-19 and dementia will be related to geography. It is clear that there are geographic differences in the experiences of the pandemic. Future research to compare temporal variability in experiences of COVID-19 and dementia with publicly available databases detailing country-level COVID-19 disease burden and mitigation strategies is needed.

### Conclusions

The purpose of this study was to use Twitter to understand the COVID-19 experiences of people with dementia and their care partners. Through an analysis of 5063 tweets, this study found that people are experiencing substantial burden from the COVID-19 pandemic. Specifically, four main issues were identified, including separation and loss, despair and abandonment, informal care partner challenges, and workforce issues experienced by formal care providers.

There is an imminent need for governments to rethink using a one-size-fits-all response to COVID-19 policy and use a collaborative approach to support people with dementia. More specifically, collaboration and partnerships are essential to developing effective COVID-19 policies to support people with dementia and their care partners. Moreover, there is a critical need for additional resources (eg, personal protective equipment, adequate staffing, safeguards, and mental health supports) to address workforce challenges and support formal care providers of people with dementia during the pandemic. Research is needed on the impact of COVID-19 from the perspective of people with dementia. Collaboration and more evidence-informed research are essential to reducing COVID-19–related mortality and improving the quality of lives for people with dementia and their care partners.

## Abbreviations

ASC: Alzheimer Society of Canada
CCNA: Canadian Consortium on Neurodegeneration in Aging
